# Impact of Covid-19 on the global orthopaedic research output

**DOI:** 10.3389/fsurg.2022.962844

**Published:** 2022-07-28

**Authors:** Milan Wolf, Stefan Landgraeber, Wolfgang Maass, Patrick Orth

**Affiliations:** ^1^ Department of Orthopaedics and Orthopaedic Surgery, Saarland University Medical Centre, Homburg, Germany; ^2^ German Research Center for Artificial Intelligence (DFKI), Saarland University, Saarbrücken, Germany

**Keywords:** COVID-19, research output, bibliometric, orthopaedic, pandemic

## Abstract

The pandemic led to a significant change in the clinical routine of many orthopaedic surgeons. To observe the impact of the pandemic on scientific output all studies published in the fields of orthopaedics listed in the Web of Science databases were analysed regarding the scientific merit of the years 2019, 2020, and 2021. Subsequently, correlation analyses were performed with parameters of regional pandemic situation (obtained from WHO) and economic strength (obtained from the World Bank). The investigations revealed that the Covid-19 pandemic led to a decrease in the annual publication rate for the first time in 20 years (2020 to 2021: –5.69%). There were regional differences in the publication rate, which correlated significantly with the respective Covid-19 case count (*r* = –.77, *p* < 0.01), associated death count (*r* = –.63, *p* < 0.01), and the gross domestic product per capita (*r* = –.40, *p* < 0.01) but not with the number of vaccinations (*r* = .09, *p *= 0.30). Furthermore, there was a drastic decrease in funding from private agencies (relative share: 2019: 36.43%, 2020: 22.66%, 2021: 19.22%), and a balanced decrease in publication output for research areas of acute and elective patient care. The Covid-19 pandemic resulted in a decline in orthopaedic annual publication rates for the first time in 20 years. This reduction was subject to marked regional differences and correlated directly with the pandemic load and was associated with decreased research funding from the private sector.

## Introduction

The pandemic caused by the Sars-Cov-2 virus, which began in 2020, led to unprecedented changes in the globalized world (1, 2). In order to at least slow the spread of the virus and thus take pressure off the medical sector, the affected countries responded with varying degrees of lockdown and social distancing (3). To ensure that patient care is maintained, many hospitals have significantly reduced elective surgeries (4).

The adopted measures also led to a change in working practices in science, as in most professional fields. In addition to absences due to illness and shortages of raw materials caused by interrupted supply chains (5), temporary closures of research facilities led to the work capacity being compromised.

In accordance with the recommendations of international and national medical associations (6), orthopaedics - as a specialty with a particularly high number of elective procedures - saw a distinct reduction in the number of surgical procedures (4, 7–10).

It remains unknown to date if and how the reduction in clinical activity as well as in scientific resources may have affected orthopaedic publication performance in the pandemic years 2020 and 2021.

Bibliometric analyses evaluate the scientific development in certain fields of research in accordance with scientific standards. By analysing the baseline data of all publications, conclusions are drawn about the quantity of published research papers (number of publications, authors, institutes, journals, etc.). This enables scientifically substantiated statements and comparisons to be made about the different years and different countries the research originated from.

We hypothesized that due to the restrictions in the professional and private environment and changes in the clinical routine, the global publication behaviour has changed depending on the pandemic load. This bibliometric study will use the most reliable and currently available Covid-19 parameters (number of cases, deaths, and vaccine doses administered) to examine the impact of the pandemic on the publication performance of individual countries.

## Methods and material

### Study design

**Figure 1:**
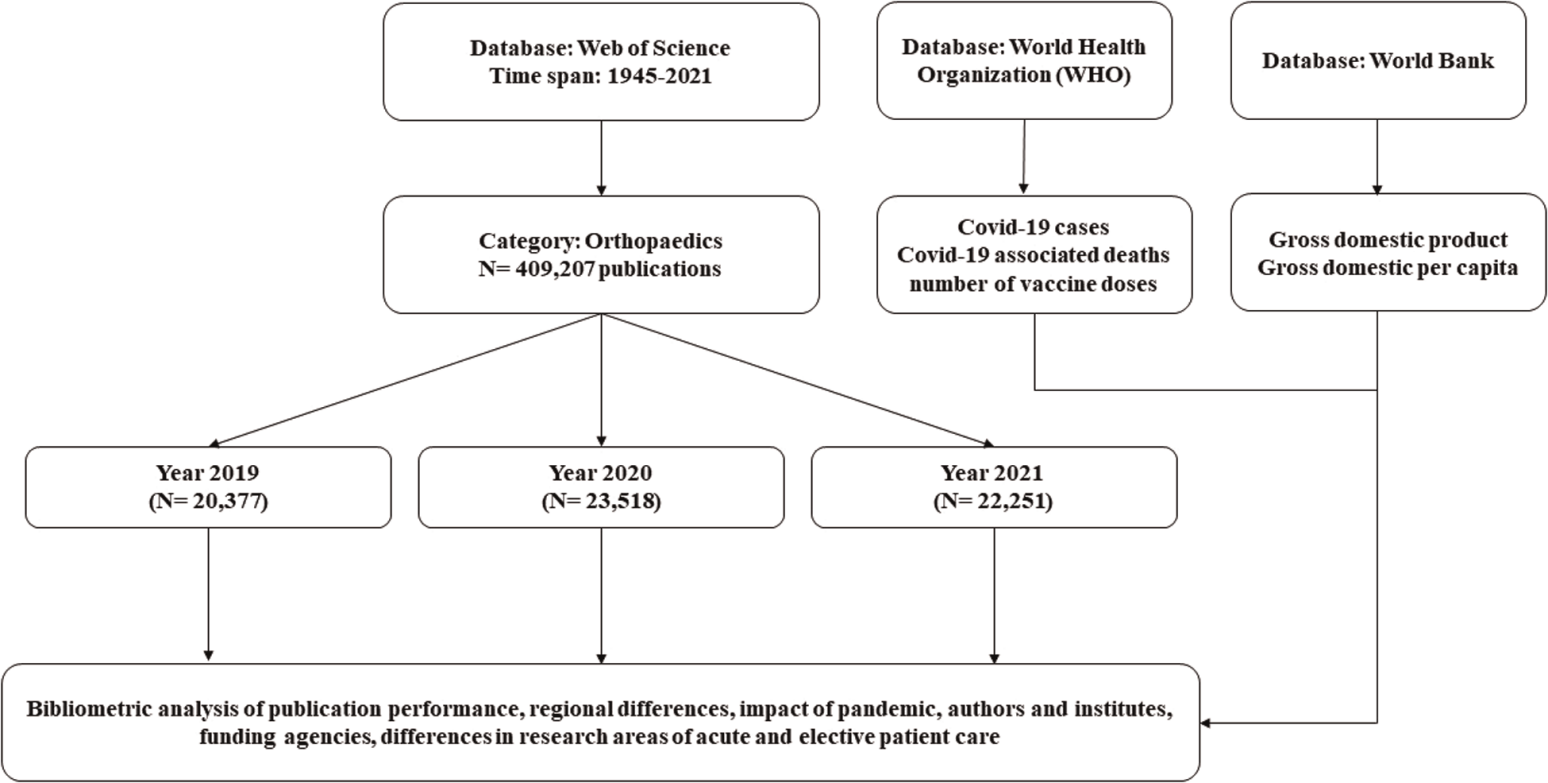
Flowchart of the study design.

In this study, the publication behaviour in the field of orthopaedics in recent years was examined. Special attention was paid to how the publication behaviour has changed due to the pandemic caused by the Covid-19 starting in 2020. All publications from 01.01.1945 until 31.12.2021 were enrolled, analysed, and compared in accordance with the methods for bibliometrics (11–16). For the years 2019 (control year), 2020 (transition year), and 2021 (pandemic year), analyses were also performed with special consideration to regional differences and correlations to the respective infection situation (Figure 1).

### Database and search strategy

The data collection was carried out using the Web of Science Core Collection of the worldwide established multi-disciplinary search platform for bibliographic database Web of Science™ (WoS) (17–20). To include as many orthopaedic publications as possible, all publications were included in the Web of Science category: orthopaedics. The assignment is made according to the subject area of the journal in which the publication was published. Categorization of journals is done in consultation with the journals by the WoS, with assignment being made immediately upon inclusion in the WoS. Journals can be assigned to additional categories over time, but the number of 6 categories cannot be exceeded. To determine whether the sub-areas of acute and elective patient care within the category orthopaedics were equally affected by the pandemic, a random sample survey was conducted. The most clinically relevant search terms “infection”, “septic”, “fracture”, “sarcoma”, and “cancer” were used and combined with boolean operators to represent acute patient care resulting in the query: TI = ((infection) OR (septic) OR (fracture) OR (sarcoma) OR (cancer)) AND WC = (Orthopedics). For research subfields of elective patient care, the most clinically relevant search terms “arthroplasty” and “arthroscopy” were used and combined with boolean operators resulting in the query: TI = ((arthroplasty) OR (arthroscopy)) AND WC = (Orthopedics).

To provide correlation with the regional infection situation, Covid-19 cases, Covid-19 associated deaths, and number of vaccine doses administered were assigned to the publication trend of the country. The current data were obtained from the World Health Organization database (21).

To ensure correlation with the countries' gross domestic product (GDP), current GDP per capita (GDP/capita) was taken from the World Bank database (22). The values of GDP/capita were given in US$.

### Analysis

After the search process for the category orthopaedics and the topics of acute and elective patient care, the corresponding publications from the years 2019, 2020, and 2021 were selected in each case. A further analysis was carried out for the respective years. For this purpose, the integrated bibliometric Web of Science analysis function was used after the search process. The total number of publications, the publications of the individual countries, the institutes, the authors, and their respective publication numbers as well as the funding agencies of the publications were identified by the Web of science analysis function and transferred to an Excel table (Microsoft Corporation, Redmond, WA). Further statistical processing was performed using GraphPad PRISM v. 9.3.1. (Graphpad Software, Inc, San Diego, CA). To investigate a possible correlation, Pearson correlation and a subsequent two-tailed significance test were performed.

Due to the large amount of data and the necessity for a manual analysis, the top 100 agencies were identified based on their number of fundings. These agencies were then assigned to either the private sector (companies) or the non-private sector (governmental, non-profit organizations, and non-governmental organizations (NGOs)) according to their economic background. Journals were assigned to a region according to the Web of Science database.

## Results

### Changes over time in publication performance

**Figure 2:**
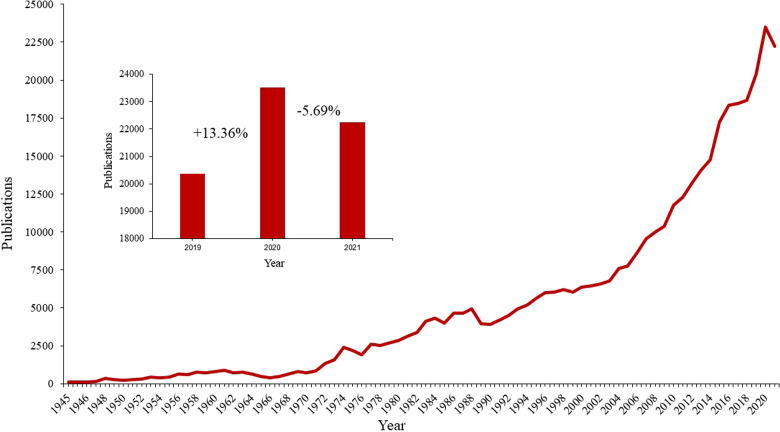
Orthopaedic publication rate over the course of time. The graph inside the figure depicts the publication rate in the years 2019, 2020 and 2021; percentages indicate the comparison with the precedent year.

Since the first record in the database in 1945, the publication rate has been steadily increasing. In the first decades, the publication rate was overall increasing, but often subject to relevant annual fluctuations. Since 1999, there has been a year-on-year increase in the publication rate in every single year. In 2021, for the first time since 1988, there was a decrease in the annual publication rate of more than 5% compared to the previous year. Specifically, there was a 5.69% decrease in publication output in 2021 compared with 2020. If the publication rate had hypothetically increased at the same rate as in 2020 (2020: 13.36%), 25,392 publications would have been expectable in 2021. This leads to a theoretical non-publication of 7,549 publications (Figure 2). The number of participating journals has changed only slightly during the period under consideration. In 2019, 127 journals were listed in the Web of Science category Orthopaedics. In 2020 and 2021, one additional journal was added to the database (128 journals). The number of journal publications was heterogeneous at the country level but decreased overall (–5.37% ± 26.73).

Due to the constant number of journals, no corresponding manipulation of the publication rate can be suspected.

### Regional differences

**Figure 3:**
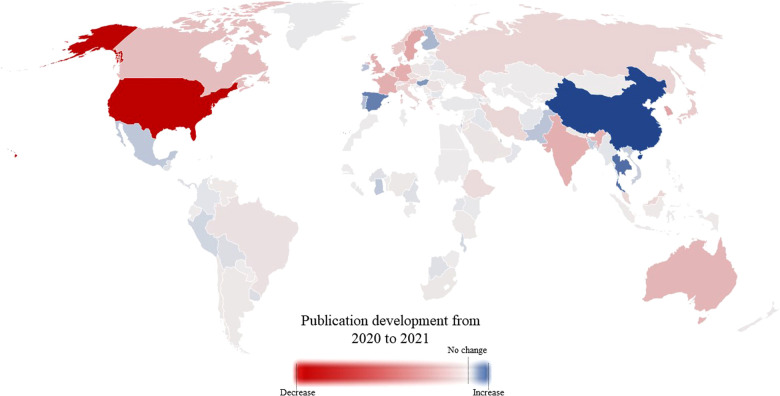
Heat map indicating the change in publications from 2020 to 2021. The colour gradient reflects alterations in the publication rate from decrease (red) to increase (blue). Countries that are marked white did not compete in orthopaedic research in the years 2019, 2020, and 2021. The legend at the bottom depicts the gradient, whereby the dotted line to the right of the centre represents no change in the publication rate.

In the years 2019 to 2021, the United States achieved the most publications followed by China, United Kingdom, and Germany. Among the most relevant publishing countries (more than 100 publications annually), there was a heterogeneous distribution of the publication rate with a decrease in the publication rate in the pandemic year 2021 in almost all countries except for some exceptions: China (+1.76%), Turkey (+0.15%), Spain (+6.49%), Thailand (+20.57%), Finland (+8.88%), Israel (+6.13%) and Ireland (+7.74%) were the only countries to increase their annual publication rate in 2021. Sweden (–35.96%), Norway (–36.42%) and Greece (–33.05%) showed the largest decrease in annual publication performance (Figure 3).

In terms of absolute publication output, the largest decrease in publications from 2020 to 2021 occurred in Europe (EU) followed by North America (NA), Asia (AS), Oceania (OC), Africa (AF), and South America (SA) (EU: −813 publications, NA: −529, AS: −307, OC: −97, AF: −16, SA −11). In relative terms (publication trend/total number of publications), the largest decrease in publications in 2021 occurred in Oceania (OC: −10.10%, EU: −9.13%, NA: −6.12%, AF: −4.04%, AS: −3.98%, SA: −1.66%).

According to the World Health Organization (21), 298,956,196 infections occurred in all countries involved in orthopaedic research, with 5,403,062 consecutive deaths. In addition, 8,887,127,085 vaccinations have been given (January 15, 2022).

### Pandemic load and the publication performance

When considering all participating countries, there was a strong correlation between the number of Covid-19 cases and Covid-19-associated deaths with the decrease in publications (Covid-19 cases: *r* = −.77, *p* < 0.01, Covid-19 deaths: *r* = −.63, *p* < 0.01). In addition, there was a moderate correlation between GDP/capita and publication rate (*r* = −.40, *p* < 0,01). There was no significant correlation between the number of applied vaccines and publication rate (*r* = .09, *p* = 0.30).

Among the most relevant countries (regularly over 100 publications per year, n= 30 countries), there was a similar Covid-19 associated effect on publication rate (Covid-19 cases: *r* = −.74, *p* < 0.01, Covid-19 deaths: *r* = −.62, *p* < 0.01; GDP/capita: *r* = .33, *p* = 0.08; vaccine: *r* = −.04, *p* = 0.85).

**Figure 4:**
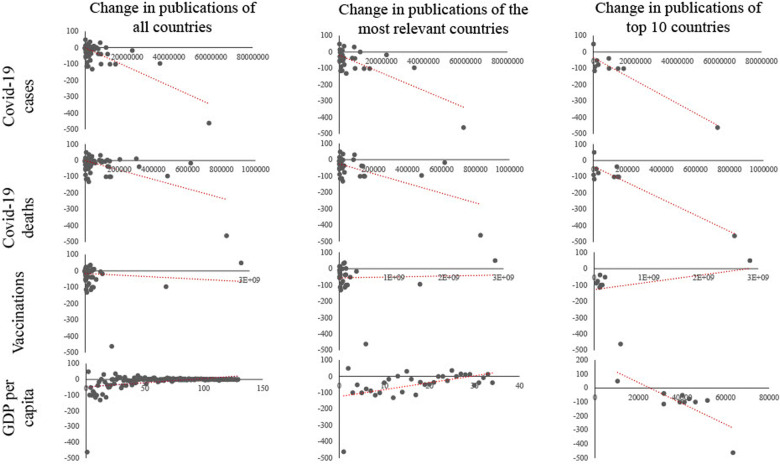
Correlation analysis between pandemic load and publication rate. The first column represents all involved countries. The second column represents the most relevant countries (annually publication rate above 100, *n* = 30). The third column represents only the top 10 countries (measured by the publication rate in 2021). The first line depicts the correlation with Covid-19 cases, the second line with Covid-19 deaths, the third line with administered Covid-19 vaccinations, and the fourth line with the country's gross domestic product per capita (GDP/capita).

Among the 10 countries with the highest publication rates, these relationships were even more pronounced, and in additional there was a weak correlation between vaccine doses administered and publication rates (Covid-19 cases: *r* = −.94, *p* < 0.01, Covid-19 deaths: *r* = −.93, *p* < 0.01; GDP/capita: *r* = −.78, *p* < 0.01**; vaccine: *r* = .29, *p* = 0.41) (Figure 4).

Related to the publications of the journals, there was a moderate correlation related to the publication rate of countries and a low correlation to the number of Covid-19 cases (Covid-19 cases: *r* = −.21, *p* = 0.39, publication rate of countries: *r* = .54, *p* = 0.01).

### Authors and institutes

In 2020, most institutes and authors participated in orthopaedic research (institutes: 2019: 20,377, 2020: 23,518, 2021: 22,251; authors: 2019: 57,254, 2020: 63,309, 2021: 63,242). In proportion to the absolute publication output, however, there was no relevant difference in participating institutes (institutes/publication: 2019: 0.59, 2020: 0.57, 2021: 0.61; authors/publication: 2019: 2.81, 2020: 2.69, 2021: 2.84) (Table 1).

**Table 1:** Development of participating authors, institutes, and private funding between 2019 and 2021.

	Number of authors	Number of institutes	Private funding
2019	57,254	20,377	36.43%
	↓ +9.59%	↓ +13.59%	↓ −13.77%
2020	63,309	23,581	22.66%
	↓ −0.11%	↓ +5.98%	↓ −3.44%
2021	63,242	22,251	19.22%

The first column shows the history of the authors involved. The second column depicts the course of the participating institutes. The third column indicates the course of the funding by private companies. The percentages between the years show the rate of year-to-year changes.

### Funding agencies

In the three years under consideration, there was no relative change in the number of funding agencies involved (funding agencies/publication: 2018: 0.27, 2020: 0.26, 2021: 0.26). The analysis of the 100 most relevant funding agencies (measured by the number of fundings made in 2021) showed a significant decrease in funding by private agencies since 2019 (share of funding from the private sector: 2019: 36.43%, 2020: 22.66%, 2021: 19.22%) (Table 1).

### Change in research areas of acute and elective patient care

The two research areas of acute and elective patient care each experienced a decrease in publication output, although this decrease was below the overall decrease in orthopaedic research (publications of acute patient care: 2019: 2,973; 2020: 3,575, 2021: 3,507, decrease of 1.90% from 2020 to 2021; publications of elective patient care: number of publications: 2019: 2,350; 2020: 2,876, 2021: 2,823, decrease of 1.84% from 2020 to 2021)

## Discussion

In this study, we were able to show for the first time the impact of the Covid-19 pandemic on the orthopaedic publication performance. In 2021, for the first time in over 20 years, there was a significant decline in annual publications compared to the respective previous year. This decline in publication output varied by region and was dependent on the number of Covid-19 infections and Covid-19 associated deaths and was associated with reduced funding by private agencies (companies).

Since the beginning of the database in use in 1945, there has been a steady increase in annual publications. Causes for the increase in orthopaedics as well as in other specialties are inter alia increasing cooperation possibilities, new technical possibilities and increased publication pressure (“publish or perish”) (23–25). Considering the consistently increasing publication rate in the last 21 years, the decrease of more than 5% compared to the previous year is indeed remarkable. An exact cause for the decline cannot be determined by this study, although it is highly likely that such decrease is multifactorial in cause. The reduction in inpatient as well as outpatient procedures (26) inevitably led to reduced feasibility of clinical trials. The work capacity released by reduced surgical procedures (4, 8, 9, 27) was often used to support care (28, 29), and thus was not available for research to a large extent. The working group of Staniscuaski was also able to show that the pandemic significantly delayed even the submission of already completed work (30). Here, the authors mainly presented private reasons (care of the children and other domestic duties) that led to the delays. A large number of other studies were also able to show increased psychological stress due to the pandemic and consequent isolation among clinically active physicians (31–33), which inevitably affects individual research capacity.

The number of authors participating in orthopaedic research changed only slightly (−0.11%) during the pandemic. In fact, more authors participated in publications relative to the absolute number of publications. This suggests that the decrease in publications is rather due to a lower number of research projects. Studies have already shown that the pandemic led to a significant decrease in new Covid-19 independent research projects (34, 35), mainly caused by a reduced possibility of data acquisition (36). Furthermore, there was understandably an increase in Covid-19 related research (37). This may have also led to a shift in resources, which may further disadvantage orthopaedic research. Our work did not address differences of race, gender, or sociocultural nature. However, profound changes can be expected in these areas as well, because in other fields of research, the Covid-19 pandemic aggravated pre-existing gaps in this regard notably (34, 35, 38–41).

We identified in this study direct correlations between the number of Covid-19 cases and Covid-19-related deaths and the number of publications. In most cases, the increased pandemic burden caused more extensive regional restrictions. Thus, higher Covid-19 cases and deaths led to a more prominent reduction in publications in 2021. Interestingly, this observation was not true for all countries and in particular, Spain, Thailand, Finland, Israel, and Ireland were able to increase their publication performance compared to the previous year. China was also able to increase its publication rate, although the growth compared to the previous year was significantly lower (29.65% vs. 1.76%). The partly pronounced differences in the publication rate are probably also based on the different approaches of the individual countries (42, 43). In terms of health care policy, in addition to these differences in the regional strategy, the respective chronological course of the restrictions or their level of severity might also play a role. A sufficiency correlation would certainly be useful to shed more light on these crucial aspects but is currently not feasible due to the partly heterogenous data situation.

Notably, there has been a decline in publication performance in both research areas of acute and elective patient care. Although the pandemic and the resulting restrictions in everyday life (e.g. commuting, decreased sports activities, and other leisure activities) (44, 45) resulted in fewer traumatological hospitalizations, elective patient care came to an almost complete standstill at times (10, 46). Thus, the subject-related workload does not seem to have had a relevant effect on publication performance. Nevertheless, there was a difference between the relative decrease in publications of acute and elective patient care and the absolute publication rate in orthopaedics. This indicates that there are important research areas within orthopaedics with a particularly stronger decline in publications, such as basic science or health service research.

In addition to the marked impact of the pandemic on the publication output, this bibliometric study also reveals its impact on global economics. The epidemic resulted in a pronounced decrease in research funding by private companies. This is most likely to be explained by the financial pressures caused by pandemic (47). Governmental funding is often long-term and can be accessed over a long period of time, whereas private companies mostly provide short- and mid-term project-related funding. In addition, private companies usually support clinical projects that can be expected to have a certain added value for the interests of the companies. The significant reduction of clinical activities in orthopaedics leads not only to increased financial pressure but also to reduced private research funding. The corresponding near 50% decrease of the private sector’s participation in financial research support inevitably leads to a reduced number of clinical and experimental studies, some of which are cost intensive. Further ccontributing factors to the decline in publication rates may be increased psychosocial stress, the reduced number of Covid-19 independent research projects, a reduced opportunity for data acquisition du to contact restrictions and a break in the supply chain.

In addition to the above-mentioned factors at the researcher level, there has also been a decline in the number of publications in the individual journals. The editors and reviewers of the journals are also suffering from the effects of the pandemic. This inevitably leads to longer internal processes and delays in the time it takes for an article to be accepted.

Bibliometric studies are generally subject to certain limitations. Even though the Web of Science databases are among the most comprehensive databases, not all publications are represented and there are deficits especially with regard to non-English publications (18). To include more publications, different databases (e.g. Embase and Medline) would have to be used, which requires the use of external software, which in turn are subject to their own limitations. Affiliations to nations are determined by the nationality of the first author, possibly reducing multicentre studies to this one nation. Due to the large amount of data and the need for manual analysis, the evaluation was limited to the 100 most influential funding organizations. This allows a good overview but no absolute statements. The research area of elective and acute patient care was examined using a selection of common keywords that represent the subfields well but do not fully cover them. Despite these limitations, this bibliometric study allows the analysis of all publications in the field of orthopaedics and thus provides an all-encompassing overview and comparison of the pandemic years.

## Conclusion

The Covid-19 pandemic resulted in a decline in the annual publication rate for the first time in over 20 years. The reduction was subject to marked regional differences and correlated directly with the number of Covid-19 cases and consecutive deaths. The change in publication behaviour was equally pronounced in acute and elective patient care research areas and was further associated with a decline in research funding from the private sector.

## Data Availability

The original contributions presented in the study are included in the article/Supplementary Material, further inquiries can be directed to the corresponding author/s.
